# TOP2A Amplification and Overexpression in Hepatocellular Carcinoma Tissues

**DOI:** 10.1155/2015/381602

**Published:** 2015-01-28

**Authors:** Ravat Panvichian, Anchalee Tantiwetrueangdet, Napat Angkathunyakul, Surasak Leelaudomlipi

**Affiliations:** ^1^Division of Medical Oncology, Department of Internal Medicine, Faculty of Medicine Ramathibodi Hospital, Mahidol University, Bangkok 10400, Thailand; ^2^Research Center, Faculty of Medicine Ramathibodi Hospital, Mahidol University, Bangkok 10400, Thailand; ^3^Department of Pathology, Faculty of Medicine Ramathibodi Hospital, Mahidol University, Bangkok 10400, Thailand; ^4^Division of General Surgery, Department of Surgery, Faculty of Medicine Ramathibodi Hospital, Mahidol University, Bangkok 10400, Thailand

## Abstract

Hepatocellular carcinoma (HCC) is the leading cause of cancer death in men worldwide owing to limited insights into pathogenesis and unsatisfactory efficacy of current therapies. HER2 and TOP2A genes are coamplified in breast and some other cancers. In this study, we investigated gene aberrations of HER2 and TOP2A and protein expressions of HER2, TOP2A, Ki-67, and p53 in tumor and matched nontumor tissues, as well as their associations with clinicopathological features. Gene aberrations were evaluated by FISH and protein expressions by IHC. Neither HER2 overexpression nor HER2 gene amplification was observed in both tumor tissues and matched nontumor tissues. By contrast, TOP2A overexpression was detected in 72.5% of tumor tissues but not detected in matched nontumor tissues. However, TOP2A gene amplification was not observed in both tumor and matched nontumor tissues. TOP2A overexpression was significantly associated with HCC tumor tissues (*P* < 0.001), hepatitis B surface antigen (HBsAg) in the serum (*P* = 0.004), and Ki-67 (*P* = 0.038) but not with age, tumor size, alpha-fetoprotein, TP53, and copy number of TOP2A gene and chromosome 17 centromere. In conclusion, TOP2A overexpression in HCC was not secondary to gene amplification. In addition, neither HER2 amplification nor overexpression could be used as prognostic and predictive marker in HCC.

## 1. Introduction

Liver cancer is the second leading cause of cancer death in men worldwide [1]. Among primary liver cancers, hepatocellular carcinoma (HCC) is the major histological subtype globally, with 78% of HCC attributable to hepatitis B virus (HBV, 53%) or hepatitis C virus (HCV, 25%) [2, 3]. HCC is typically an aggressive tumor arising from chronic liver disease and liver cirrhosis. Prognosis of HCC remains dismal. The majority of HCC patients are not candidates for curative therapies (surgical resection or liver transplantation) due to advanced or unresectable disease at presentation, and the available therapeutic options include local nonsurgical methods of tumor ablation and systemic therapy.

Certain oncogenes, including human epithelial growth factor receptor-2 (HER2) and topoisomerase II alpha (TOP2A), have been investigated in various cancer subtypes as targets for cancer therapy, as well as biomarkers for prediction of response. HER2 gene is a proto-oncogene located on chromosome 17 (17q11.2-q12) and encodes a 185 kDa transmembrane tyrosine kinase receptor [4, 5]. HER2 gene amplification in breast cancer has become an important biomarker for predicting response to HER2-targeted therapy [6, 7]. However, the role of HER2 gene status and HER2 protein expression in HCC has been controversial [8–15]. TOP2A gene is located on chromosome 17 (17q21-q22), at about 700 kb telomeric to the HER2 gene locus [16]. TOP2A gene encodes a 170 kDa nuclear enzyme that controls DNA topological structure, chromosome segregation, and cell cycle progression [17–19]. TOP2A protein is targeted by several chemotherapeutic agents such as anthracyclines (doxorubicin, epirubicin) and epipodophyllotoxins (etoposide, teniposide). TOP2A gene is coamplified with HER2 gene in approximately 35% of HER2-amplified breast cancers, and TOP2A coamplification, not HER2 amplification, is the predictive marker of an incremental response to anthracycline-based chemotherapy in HER2-amplified breast cancers [7, 20–23]. TOP2A overexpression, that is, increased level of TOP2A mRNA and protein, has been detected in HCC [24, 25], but it remains unclear whether TOP2A overexpression in HCC has arisen from TOP2A gene amplification. In addition, no previous study has investigated whether TOP2A and HER2 coamplification exists in HCC. Therefore, to determine the association between TOP2A/HER2 amplification and TOP2A/HER2 overexpression in HCC, we investigated gene copy number of both HER2 and TOP2A by fluorescence in situ hybridization (FISH) and protein expressions of HER2, TOP2A, as well as Ki-67, and tumor protein p53 (TP53) by immunohistochemical staining (IHC). Then, the associations of these gene status and protein expressions with clinicopathological features were statistically analyzed.

## 2. Materials and Methods

### 2.1. Tumor and Matched-Tumor Samples

Forty pairs of hepatocellular carcinoma (HCC) and nontumor tissues from the same patients were obtained by surgical resection from the operating rooms, Department of Surgery, Ramathibodi Hospital. Tissues were snap-frozen at −80°C. Frozen sections were prepared and stained with hematoxylin and eosin (H&E). The H&E stained histological slides were reviewed by an experienced pathologist. Samples were mapped with these H&E slides so that tumor and matched nontumor areas were selected for preparations of isolated nuclei and for IHC assay. This study was approved by the Ethics Committee on Research involving Human Subjects of the Faculty of Medicine, Ramathibodi Hospital, Mahidol University.

### 2.2. Preparation of Isolated Nuclei for FISH Assay

Twenty-two pairs of frozen tumor and matched nontumor tissues were available for preparation of isolated nuclei for FISH assay. Isolated nuclei were prepared by the method previously reported [26]. Briefly, selected tissue samples were incubated in 500 *μ*L 0.9% NaCl pH 1.5 for 1 h at 37°C, with occasional vortexing. Tissues suspensions were washed with PBS (phosphate buffered saline) and strained through a cell strainer (BD Falcon, USA). Isolated nuclei were fixed in Carnoy's fixative (3 : 1 methanol : acetic acid). Slide preparations were made by dropping 8 *μ*L of each nuclei suspension onto glass slides and then drying them in a 65°C oven for 15 min. The preparations were kept at −20°C until used.

### 2.3. FISH Assay

HER2 and TOP2A gene amplification were assessed by fluorescence in situ hybridization using the PathVysion HER-2 DNA probe kit and TOP2A DNA probe kit, respectively (Vysis Inc., Downers Grove, Illinois, USA), according to the procedure specified by the manufacturer with the following modifications. Briefly, slides were incubated in 2XSSC at 75°C for 20 min. The slides were then digested with pepsin (4 mg/mL in 0.9% NaCl, pH 1.5) for 5 min, dipped in water and rinsed with 2XSSC at room temperature for 5 min. The probe and target DNA were codenatured at 80°C for 5 min and hybridized in a moisture chamber overnight at 37°C. After hybridization, coverslips were gently removed and the slides were washed with 0.4XSSC/0.3%NP-40 at 73°C for 2 min. The slides were then transferred to 2XSSC/0.1% NP-40 at room temperature for 1 min. Nuclei were counterstained with DAPI II (Vysis Inc., Downers Grove, Illinois, USA). Signals were counted under a fluorescence microscope (Olympus BX61) equipped with a 100 W mercury lamp. To view signals, single bandpass filters for spectrum green, spectrum orange, and DAPI were used. At least a total number of 100 interphase nuclei were scored from most of specimens. HER2 status was determined according to the ASCO/CAP guidelines [27]. A HER2/centromere of chromosome 17 (HER2/CEP17) ratio of less than 1.8 was considered as HER2 negative (HER2 nonamplified), a HER2/CEP17 ratio between 1.8 and 2.2 as HER2 equivocal, and a HER2/CEP17 ratio higher than 2.2 as HER2 positive (HER2 amplified). TOP2A gene amplification was defined as a TOP2A/CEP17 ratio ≥2.0, whereas TOP2A deletion was defined as a TOP2A/CEP17 ratio ≤0.8. The case was considered as normal when the ratio of TOP2A/CEP17 was between 0.8 and 2.0. Gain of chromosome 17 centromere was defined as the average CEP17 ≥3 [26, 28, 29]. Images of nuclei were captured by Cytovision software (Leica, UK).

### 2.4. IHC Assay

Forty pairs of tumor and matched nontumor tissues were fixed in 10% buffered formalin and then processed and embedded in paraffin. Serial 4-micron sections were cut and placed on positive charged slides. Slides were deparaffinized in xylene and hydrated through graded concentrations of ethanol and finally distilled water. Antigen retrieval was carried out at this stage with 10 mM citrate buffer, pH 6.0, in a pressure cooker. Sections were then processed with an UltraVision LP Value Detection System (Lab Vision, USA). Briefly, sections were blocked with Hydrogen Peroxide Block for 15 min at room temperature, followed by Ultra V Block for 10 min at room temperature. Primary antibody of each marker was applied at an optimized dilution and the incubation time, as shown in Table 1. Sections were incubated with Value Primary Antibody Enhancer for 30 min at room temperature; then, Value HRP Polymer was applied and the sections were incubated for 1 h at room temperature. DAB (3, 3′-diaminobenzidine) was used as substrate to reveal the expression of each marker. Slides were counterstained with hematoxylin and mounted in permanent mounting medium. Tissues with omission of the specific antibody were used as negative controls. Slides were scanned with the Pannoramic MIDI digital slide scanner (3DHISTECH, Hungary).

### 2.5. IHC Staining Interpretation

According to ASCO/CAP guidelines [27], HER2 expression was scored as follows: 0, no staining; 1+, weak and incomplete membranous staining in >10% of the tumor cells; 2+, weak to moderate, complete membranous staining in >10% of the tumor cells; 3+, strong, complete membranous staining in >30% of the tumor cells.

The cut-off value for tumor protein p53 (TP53) positivity was a p53 staining with positive nuclei in ≥1% of cells, regardless of the staining intensity. Ki-67 proliferation index and TOP2A expression were assessed by visual estimation of the percentage of positive nuclei in the tissues. Ki-67 proliferation index ≥10% was defined as Ki-67 positive. TOP2A expression ≥10% was defined as TOP2A overexpression by immunohistochemistry.

### 2.6. Serum Hepatitis B Surface Antigen (HBsAg) Assay

Chemiluminescent microparticle immunoassays (CMIA) for the qualitative detection of hepatitis B surface antigen (HBsAg) in serum from the patients were performed using ARCHITECT HBsAg Qualitative II assay (Abbot Laboratories, Illinois, USA).

### 2.7. Serum Alpha-Fetoprotein (AFP) Assay

Electrochemiluminescence immunoassays (ECLIA) for the in vitro quantitative determination of alpha-fetoprotein (AFP) in serum from the patients were performed using the AFP kit with a cobas e601 analyzer (Roche Diagnostics Limited GmbH, Mannheim, GM).

### 2.8. Statistical Analyses

Statistical analyses were performed with SPSS v.11.5 (SPSS Inc., Chicago, Illinois, USA). Association between TOP2A expression and other clinicopathological variables was determined using a chi-square test (*χ* ^2^ test). The *P* values less than 0.05 were considered statistically significant.

## 3. Results

### 3.1. Clinicopathological Features

Forty patients (35 men, 5 women; age range 35–94 years, median 51 years) with resectable hepatocellular carcinoma were included in this study. Clinicopathological features are summarized in Table 2. Sixty-five percent of patients had tumor sizes greater than or equal to 5 cm. Presence of HBsAg in the serum was detected in 70% of patients. Thirty-two percent of patients presented with serum alpha-fetoprotein (AFP) values greater than or equal to 500 ng/mL. Positive TP53 and KI-67 expression in HCC tissues were detected in 45% and 75%, respectively.

### 3.2. Association of HER2 Gene Status and Protein Expression

**Figure 1:**
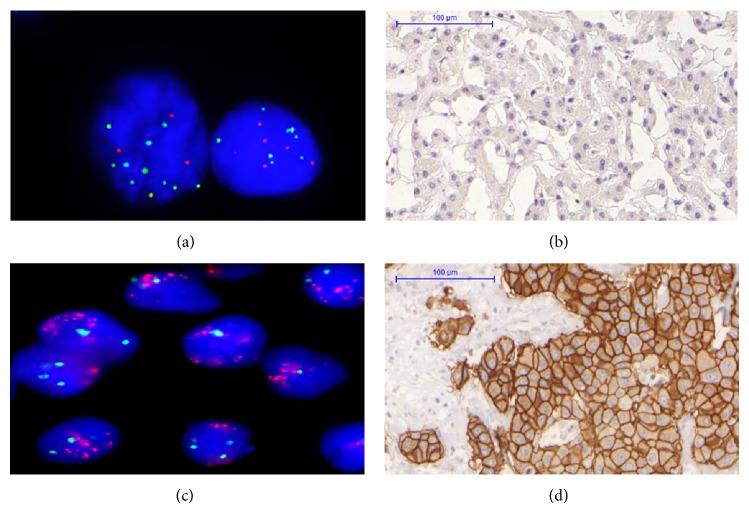
Representative HER2 (red signal) and CEP17 (green signal) copy number detected by FISH and HER2 protein expression detected by IHC. (a) CEP17 gain with nonamplified HER2 in case 34, original magnification ×1000. (b) HER2 negative expression in case 34. (c) HER2 amplification in breast cancer tissue (positive control, original magnification ×1000). (d) HER2 protein expression positive 3+ in breast cancer tissue (positive control, same case as (c)).

HER2 protein overexpression was investigated in 40 pairs of tumor and matched nontumor tissues; HER2 gene amplification was investigated in 22 pairs of the tissues. The results showed that neither HER2 overexpression (*n* = 40 pairs) nor HER2 gene amplification (*n* = 22 pairs) was detected in tumor tissues and matched nontumor tissues. In addition, HER2 gene deletion (HER2/CEP17 ratio ≤0.8) was detected in 9% (2/22) of tumor tissues, and gain of chromosome 17 centromere (CEP17 ≥ 3) was observed in 50% (11/22) of tumor tissues. Representative CEP17 gain and its negative HER2 expression in tumor tissue are shown in Figure 1.

### 3.3. Association of TOP2A Gene Status and Protein Expression

**Figure 2:**
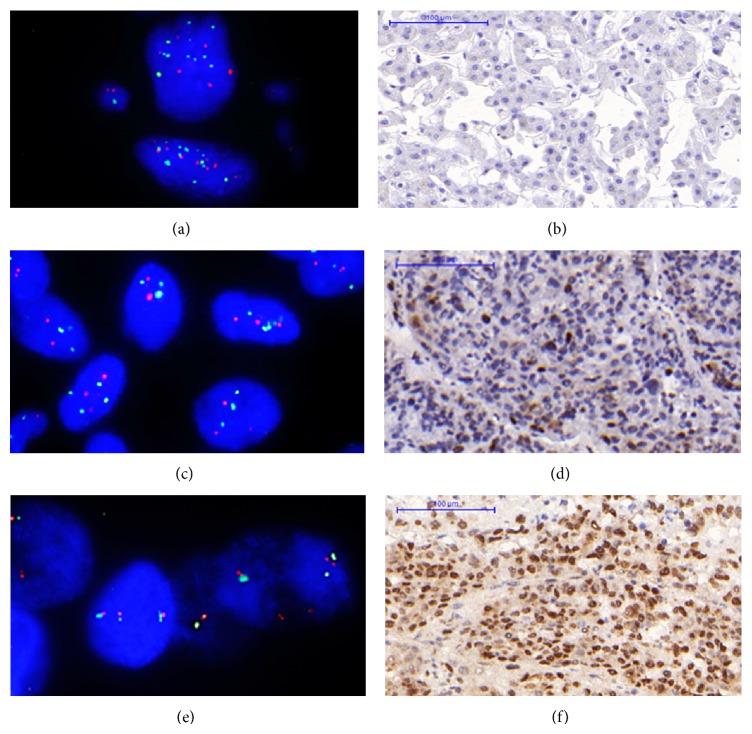
The disconcordance between TOP2A gene (TOP2A, red signal; CEP17, green signal) copy number detected by FISH and TOP2A protein (brown signal) expression detected by IHC in cases of HCC. (a) Case 34: FISH result showed TOP2A deletion (ratio = 0.65, original magnification ×1000) and negative TOP2A expression as shown in (b). (c) Case 32: FISH result showed TOP2A deletion (ratio = 0.64, original magnification ×1000) and TOP2A overexpression (30%) as shown in (d). (e) Case 25: FISH result showed normal TOP2A (ratio = 1.07, original magnification ×1000) and TOP2A overexpression (90%) as shown in (f).

TOP2A protein overexpression was investigated in 40 pairs of tumor and matched nontumor tissues; TOP2A gene amplification was investigated in 22 pairs of the tissues which also used for HER2 gene amplification study. However, the results of TOP2A gene status are available only in 20 tumor tissues and 18 matched nontumor tissues. TOP2A overexpression was detected in 72.5% (29/40) of tumor tissues but was not detected in nontumor tissues. However, TOP2A gene amplification was not detected in both tumor tissues and matched nontumor tissues. In addition, TOP2A gene deletion (TOP2A/CEP17 ratio ≤0.8) concurrently with HER2 gene deletion (HER2/CEP17 ratio ≤0.8) was observed in 10% (2/20) of tumor tissues. One of the tumor tissues with TOP2A gene deletion showed TOP2A overexpression (Case 32), whereas the other tumor tissue showed no expression of TOP2A (Case 34). The discordance between TOP2A gene copy number and TOP2A protein expression is shown in Figure 2. Gain of chromosome 17 centromere (CEP17 ≥ 3) was detected in 60% (12/20) of tumor tissues.

### 3.4. Association of TOP2A Protein Expression and Other Clinicopathological Features

TOP2A overexpression was detected in 72.5% (29/40) of tumor tissues and significantly associated with HCC tissues (*P* < 0.001); in other words, it was only found in HCC tumor tissues. In addition, TOP2A overexpression in tumor tissues was also significantly associated with HBsAg in the serum (*P* = 0.004) and Ki-67 expression (*P* = 0.038). Nevertheless, there was no significant association of TOP2A overexpression with age, tumor size, AFP, TP53 expression, CEP17 copy number, and TOP2A gene copy number, as demonstrated in Table 3.

## 4. Discussion

In the present study, 65% of patients had tumor size ≥5 cm indicating that these HCC patients were diagnosed at a later stage, and also 70% of the patients had HBV-associated HCC, as indicated by the presence of HBsAg in the serum. We also found that these HCC tissues frequently expressed TP53 (45%) and Ki-67 (75%), similar to previous reports [30–34]. It has been reported that the prevalence of HBV and the frequency of TP53 expression are higher in Asian HCC patients than in American HCC patients [30].

HER2 amplification and overexpression are found in several cancers including breast, ovarian, endometrial, and pancreatic carcinomas [35]. HER2 overexpression is usually a result of HER2 gene amplification. In addition, HER2 amplification as well as overexpression is associated with poor prognosis in breast cancer and has become an essential biomarker predictive for the response to HER2-targeted therapy [6, 7]. In contrast, the investigation of HER2 gene status and expression in HCC has yielded varying results. Although some studies reported that HER2 is amplified and overexpressed in HCC tissues [8, 9, 12–15], Xian et al. [14] and Bacaksiz et al. [15] concluded that overexpression as well as amplification of HER2 is uncommon in HCC. Hsu et al. [10] also reported that HER2 overexpression is rare in patients with advanced HCC. However, our results showed that HER2 was neither amplified nor overexpressed in both tumor tissues and matched nontumor tissues, being consistent with the result of Vlasoff et al. [11]. Furthermore, our results showed that the average copy number of chromosome 17 centromere (CEP17) was significantly higher in tumor tissues than in matched nontumor tissues (3.55 ± 1.99 versus 2.07 ± 0.22, *P* < 0.01), but the gains of CEP17 in HCC were not associated with HER2 overexpression. This is consistent with our previous study in breast cancer tissues that, in the absence of HER2 amplification, gains of CEP17 do not have a significant effect on HER2 expression [26]. Because of its rarity or absence, we concluded that neither HER2 amplification nor overexpression could be a commonly used prognostic and predictive marker in hepatocellular carcinoma.

TOP2A gene encodes a 170 KDa nuclear enzyme controlling DNA topological structure and is frequently coamplified with HER2 gene in breast cancer and bladder cancer [22, 36–39]. TOP2A gene amplification and TOP2A deletion could be found in 35% and 5% of HER2-amplified breast cancer, respectively; however, only TOP2A gene deletion could be detected in 3% of HER2 nonamplified breast cancer [22]. It has been reported that coamplification of HER2 and TOP2A in breast cancer is associated with sensitivity to anthracycline [7, 20–23, 40]. TOP2A coamplification in HER2-amplified breast cancer might cause an increased sensitivity to TOP2A inhibitors by inducing the overexpression of the TOP2A protein [22, 37]. Furthermore, TOP2A expression has been reported to be a valuable prognostic marker for tumor advancements, recurrences, and predictor of poorer survival in small cell lung cancer, ovarian, colon, breast, and prostate cancers, as well as nasopharyngeal carcinoma [41–46]. TOP2A overexpression is a strong indicator of poor prognosis in prostate cancer and in nasopharyngeal carcinoma as well as in the subgroup of HER2 negative breast cancer [45–48]. A few studies have found TOP2A overexpression in HCC [24, 25]. However, it has not yet been determined whether TOP2A overexpression in HCC has arisen from TOP2A gene amplification.

In this study, TOP2A was significantly overexpressed in HCC tumor tissues (*P* < 0.001), but TOP2A gene amplification was not detected in these tissues, as well as no significant association between TOP2A protein expression and TOP2A gene copy number. Based on these results, we have demonstrated for the first time that TOP2A overexpression in HCC did not arise from TOP2A gene amplification. Previously, some studies in prostate cancer, breast cancer, and gastric carcinomas also reported that TOP2A overexpression is not closely correlated with TOP2A amplification, in contrast to the strong correlation of HER2 overexpression with HER2 amplification [45, 49–52]. In addition, Schindlbeck et al. concluded that TOP2A protein expression might rather be the target of antracycline independent from gene copy number [50]. Isaacs et al. have shown that TOP2A is highly regulated at transcriptional and translational level [18, 19], suggesting that TOP2A overexpression may arise from aberration at the transcriptional or translational level. Furthermore, Srikantan et al. have recently identified the RNA-binding protein HuR and the microRNA miR-548c-3p as the crucial mediators that control TOP2A expression levels and determine the effectiveness of doxorubicin [53].

To the best of our knowledge, this report is the first study to show a statistically significant association between TOP2A overexpression and HBsAg in the serum (*P* = 0.004). However, this association needs to be validated due to the relatively small sample size of this study and most of the HCC patients in the present study are HBsAg positive. A significant association between TOP2A overexpression and Ki-67 expression in HCC tissues (*P* = 0.038) has also been observed in the current study. Ki-67 protein is a human nuclear protein expressed only in proliferating cells during all of the active phases of the cell cycle (G1, S, G2, and M phases) but not in the resting cells (G0) [54], while TOP2A expression is undetectable until late S phase, peaked in G2-M phase, and decreased as the cells completed mitosis [55, 56]. Both Ki-67 and TOP2A expression can be used as molecular biomarkers of cell proliferation in various cancers. TOP2A expression is strongly correlated with Ki-67 expression in breast cancer [47–49]. Moreover, Ki-67 expression has been found to correlate with tumor growth rate and poor prognosis in HCC [33, 34].

TOP2A overexpression in HCC has prognostic significance. Watanuki et al. found that TOP2A overexpression in HCC appears to be linked with a potentially aggressive tumor phenotype and cancer-related death [24]. Furthermore, Wong et al. reported that TOP2A overexpression in HCC correlates with early age onset, shorter survival times, and resistance to doxorubicin-based chemotherapy [25]. Therefore, novel TOP2A-targeted chemotherapeutic agents with better efficacy are urgently needed. Mechanisms of doxorubicin-resistance are thought to involve the induction of multidrug resistance (MDR) mediated by ATP-dependent drug efflux pumps, in particular, ABCB1 (MDR1, Pgp) and ABCC1 (MRP1), as well as the concomitant rise in the level of topoisomerase I. Thus, detection of TOP2A overexpression in HCC tissues might help select appropriate patients for early clinical trials of the novel TOP2A-targeted therapeutic agents, which should include a new class of topoisomerase II inhibitors with potential antimultidrug resistance capabilities [54] and dual topoisomerase I and II inhibitors [55].

In summary, we found that (1) HER2 was neither amplified nor overexpressed in both HCC tumor tissues and nontumor tissues; (2) TOP2A overexpression in HCC tumor tissues did not arise from TOP2A gene amplification and was independent from HER2 gene amplification or overexpression; and (3) TOP2A overexpression was significantly associated with HBsAg in the serum, as well as with Ki-67 expression. Thus, HCC patients with TOP2A overexpression might be potential candidates for novel TOP2A-targeted therapeutic trials.

## Supplementary Material

The supplementary material provides the result of FISH assay showing copy number of HER2 gene, TOP2A gene, and CEP 17 in 22 pairs of HCC and matched non-tumor tissues.

## Figures and Tables

**Table 1:** Source, dilution, antigen retrieval method, and incubation time of different biomarkers.

Marker	Source	Host	Dilution	Antigen retrieval	Incubation time
Her-2	SP3 (Lab Vision, USA)	Rabbit	1 : 400	10 mM citrate buffer pH 6.0, pressure cooker	40 min, RT
Topo2*α*	Ki-SI (Lab Vision, USA)	Mouse	1 : 400	10 mM citrate buffer pH 6.0, pressure cooker	30 min, RT
P53	Y5 (Lab Vision, USA)	Rabbit	1 : 100	10 mM citrate buffer pH 6.0, pressure cooker	30 min, RT
Ki-67	SP6 (Lab Vision, USA)	Rabbit	1 : 200	10 mM citrate buffer pH 6.0, pressure cooker	30 min, RT

**Table 2:** Clinicopathological features of HCC patients (*n* = 40).

Patients	*n* (%)
Age (years)	
<50	18 (45.0)
≥50	22 (55.0)
Range	35–94
Mean	52.5
Median	51
Sex	
Male	35 (87.5)
Female	5 (12.5)
HBsAg	
Negative	12 (30.0)
Positive	28 (70.0)
AFP	
<500 ng/mL	24 (60.0)
≥500 ng/mL	13 (32.5)
Unknown	3 (7.5)
Tumor size	
<5 cm	14 (35.0)
≥5 cm	26 (65.0)
TP53 expression	
Negative	21 (52.5)
Positive	18 (45.0)
Unknown	1 (2.5)
Ki-67 expression	
<10%	9 (22.5)
≥10%	30 (75.0)
Unknown	1 (2.5)

HBsAg: hepatitis B surface antigen; AFP: alpha fetoprotein; HCC: hepatocellular carcinoma; TP53: tumor protein p53.

**Table 3:** Association between TOP2A overexpression and other variables.

Variables	TOP2A overexpression^†^		*P* ^*^
	Negative (IHC < 10%)	Positive (IHC ≥ 10%)	
Tissues			
Nontumors	40 (100%)	0 (0%)	<0.001
Tumors	11 (27.5%)	29 (72.5%)	
Age			
<50	4 (22.2%)	14 (77.8%)	0.499
≥50	7 (31.8%)	15 (68.2%)	
Tumor size			
<5 cm	11 (78.6%)	3 (21.4%)	0.528
≥5 cm	18 (69.2%)	8 (30.8%)	
AFP			
<500	7 (29.2%)	17 (70.8%)	0.919
≥500	4 (30.8%)	9 (69.2%)	
HBsAg			
Negative	7 (58.3%)	5 (41.7%)	0.004
Positive	4 (14.3%)	24 (85.7%)	
TP53 expression			
Negative	7 (33.3%)	14 (66.7%)	0.442
Positive	4 (22.2%)	14 (77.8%)	
Ki-67 expression			
<10%	5 (55.6%)	4 (44.4%)	0.038
≥10%	6 (20.0%)	24 (80.0%)	
CEP17 copy number			
<3 copy	2 (25.0%)	6 (75.0%)	1.000
≥3 copy	3 (25.0%)	9 (75.0%)	
TOP2A copy number			
<3 copy	1 (16.7%)	5 (83.3%)	1.000
≥3 copy	4 (28.6%)	10 (71.4%)	

TOP2A overexpression = TOP2A (IHC) ≥10%.

^†^Result given as *n* (%).

^*^Chi-square (*χ* ^2^) test.
