# Supplementary material for: TOP2A Amplification and Overexpression in Hepatocellular Carcinoma Tissues
Source: Biomed Res Int. 2015 Jan 28;2015:381602. doi: 10.1155/2015/381602 (PMC4324886; doi:10.1155/2015/381602)
Supplement: Supplementary file 1 — The supplementary material provides the result of FISH assay showing copy number of HER2 gene, TOP2A gene, and CEP 17 in 22 pairs of HCC and matched non-tumor tissues. [file 381602.f1.pdf]

Table 1. HER2 gene and CEP 17 copy number in 22 pairs of HCC tissues and matched non-tumor tissues

		**Matched non-tumor tissues**			**HCC tissues**		
No.	Case	Cep17	Her-2	Ratio	Cep17	Her-2	Ratio
1	7	1.83	2.19	1.19	2.79	2.8	1.00
2	10	2.39	2.75	1.15	6.65	6.87	1.03
3	14	1.94	2	1.03	1.98	2.04	1.03
4	15	2.01	2.09	1.03	3.55	3.66	1.03
5	17	1.95	2.43	1.24	3.78	3.85	1.01
6	19	1.93	1.99	1.03	3.54	3.63	1.02
7	20	2.55	2.6	1.01	2.84	3.18	1.11
8	21	1.97	2.03	1.03	4.32	4.46	1.03
9	24	2.18	2.21	1.01	2.25	2.29	1.01
10	25	2.27	2.39	1.05	2.57	2.58	1.00
11	29	2.6	2.98	1.14	2.71	2.74	1.01
12	31	2	2.17	1.08	3.42	3.56	1.04
13	32	2.07	2.36	1.14	4.53	3.03	0.66
14	33	1.9	2.15	1.13	2.06	2.12	1.02
15	34	1.96	2.18	1.11	11.06	8.09	0.73
16	35	1.91	2.07	1.08	2.45	3.52	1.43
17	36	1.96	2.18	1.11	2.82	3.1	1.09
18	37	1.8	1.95	1.08	4.12	4.12	1.00
19	38	2.24	2.47	1.10	2.1	2.1	1.00
20	39	2.1	2.35	1.11	3.24	3.45	1.06
21	40	2.04	2.61	1.27	3.31	3.61	1.09
22	42	1.95	2.01	1.03	2.04	2.03	0.99

Cep 17 = copy number of chromosome 17 centromere by FISH; CEP17 ≥3 = Gain of chromosome 17 centromere;

Her-2 = copy number of HER2 by FISH

Ratio = HER2/Cep17 ratio; Ratio ≤ 0.8 = HER2 gene deletion

0.8 < Ratio < 1.8 = HER2 non-amplified

1.8 ≤ Ratio <2.2 = HER2 equivocal

Ratio ≥ 2.2. = HER2 amplified (positive)
