# Supplementary material for: TOP2A Amplification and Overexpression in Hepatocellular Carcinoma Tissues
Source: Biomed Res Int. 2015 Jan 28;2015:381602. doi: 10.1155/2015/381602 (PMC4324886; doi:10.1155/2015/381602)
Supplement: Supplementary file 2 [file 381602.f2.pdf]

**Table2.** TOP2A gene and CEP17 copy number in 22 pair of HCC tissues and matched non-tumor tissues

| | | **Matched non-tumor tissues** | | | **HCC tissues** | | |
|---|---|---|---|---|---|---|---|
| No. | Case | Cep17 | TOP2A | Ratio | Cep17 | TOP2A | Ratio |
| 1 | 7 | 1.83 | 2.07 | 1.13 | 3.67 | 3.80 | 1.03 |
| 2 | 10 | 2.20 | 2.29 | 1.04 | 7.00 | 7.77 | 1.11 |
| 3 | 14 | 2.0 | 2.13 | 1.07 | 1.93 | 2.00 | 1.04 |
| 4 | 15 | 1.84 | 2.09 | 1.14 | 3.72 | 3.90 | 1.05 |
| 5 | 17 | 1.87 | 2.57 | 1.37 | 3.13 | 3.51 | 1.12 |
| 6 | 19 | 2.00 | 2.09 | 1.05 | 3.58 | 3.84 | 1.07 |
| 7 | 20 | 2.60 | 3.68 | 1.42 | 3.74 | 4.10 | 1.10 |
| 8 | 21 | NA | NA | NA | 4.48 | 4.8 | 1.07 |
| 9 | 24 | 2.34 | 3.03 | 1.29 | 2.21 | 2.71 | 1.23 |
| 10 | 25 | 2.00 | 2.00 | 1.00 | 2.42 | 2.59 | 1.07 |
| 11 | 29 | 2.43 | 2.92 | 1.20 | 3.29 | 3.97 | 1.21 |
| 12 | 31 | 2.34 | 2.66 | 1.14 | 4.07 | 4.07 | 1.00 |
| 13 | 32 | 1.88 | 2.20 | 1.17 | 4.76 | 3.04 | 0.64 |
| 14 | 33 | NA | NA | NA | NA | NA | NA |
| 15 | 34 | 1.97 | 2.11 | 1.07 | 12.33 | 8.00 | 0.65 |
| 16 | 35 | 1.89 | 2.00 | 1.06 | 2.66 | 4.11 | 1.54 |
| 17 | 36 | NA | NA | NA | 2.37 | 2.46 | 1.04 |
| 18 | 37 | 2.12 | 2.34 | 1.10 | 3.97 | 4.19 | 1.05 |
| 19 | 38 | 2.78 | 2.50 | 0.9 | 1.97 | 2.15 | 1.09 |
| 20 | 39 | NA | NA | NA | NA | NA | NA |
| 21 | 40 | 1.99 | 2.48 | 1.25 | 2.92 | 3.34 | 1.14 |
| 22 | 42 | 1.92 | 2.04 | 1.06 | 2.10 | 2.32 | 1.10 |

NA = not available

Cep 17 = copy number of chromosome 17 centromere by FISH; CEP17 $\geq$3 = Gain of chromosome 17 centromere;

TOP2A = copy number of TOP2A by FISH

Ratio = TOP2A/Cep17 ratio; Ratio $\leq$ 0.8 = TOP2A gene deletion

0.8 < Ratio < 2 = TOP2A non-amplified

Ratio $\geq$ 2 = TOP2A amplified (positive)
